# Building the Clinical Bridge to Support Nursing Effectiveness Science

**DOI:** 10.1155/2012/194147

**Published:** 2012-07-05

**Authors:** Kathleen Potempa, John Daly, Marita G. Titler

**Affiliations:** ^1^University of MI School of Nursing, Ann Arbor, MI 48109-5482, USA; ^2^World Health Organization Collaborating Centre for Nursing, Midwifery and Health Development, Faculty of Nursing, Midwifery & Health, University of Technology Sydney, Sydney, Australia; ^3^UMHS, University of Michigan School of Nursing, Ann Arbor, MI 48109-5482, USA

## 1. Background

Several forces in the US, including the need to control rising health care costs, are converging to require quality and safety outcomes as a factor in reimbursement for health care services.Many of the established outcomes, including nosocomial decubitus ulcers, falls with injury, medication errors, and self-care management are quality indicators sensitive to nursing care. Requirements for transparency in reporting outcomes as well as the rapid expansion of electronic health records (EHRs) are producing opportunities to study clinical care processes and outcomes as heretofore not possible. The ability of nurse researchers to define the database structures of EHRs, access these data and set forth analyses for health systems to improve care is a compelling reason to build solid relationships between academic and health care institutions. But perhaps more compelling is the opportunity to fully advance the professionalization of nursing through the unification of missions, ultimately for the betterment of patient care and the improved education of new generations of nurses. 

Other professions, especially physicians, have long experienced the benefits of uniting their tripartite missions of patient care, education, and research. They have utilized the “power” of the Academic Medical Center to fuel discovery, innovation and the rapid translation to clinical care. Medical students and postgraduate trainees benefit from this environment; their mentors are faculty with significant research programs, attending physicians responsible for patient care, leaders within the health system administrative structures driving policy, and teachers of the next generation. The opportunity of a unification model of varying types, to advance professionalization and influence of nursing in care decisions, policy, and the discovery and translation of knowledge to care practices is essential for us to leverage this “new age” where much is expected of nurses and nursing in the evolution of health care within countries and in the US, health care reform. 

The future of nursing requires that we employ a unified approach in research, education, and practice. Consequently, there is an imperative (1) for nurses to practice to the fullest extent of their education and training; (2) to improve educational systems that offer seamless progression to higher levels of education; (3) for nurses to be full partners with physicians and other healthcare professionals to transform healthcare delivery; and (4) to meet work force demands for nurses globally [1].

### 1.1. Effectiveness Science

One of the emerging sciences that will benefit from a unified academic-practice environment is “effectiveness science.” Effectiveness science, essential to improving quality and cost of healthcare, includes testing health care interventions in real world settings, advancing use of research findings by diverse populations, and training and career development for the next generation of investigators in the field. Effectiveness science uses comparisons, subgroups, and context to determine what health care treatments work for whom and in what setting. This includes comparisons of treatments as well as models of care delivery [2]. 

Effectiveness science is receiving increasing attention in healthcare [3]. The purpose of effectiveness research is to help consumers, clinicians, purchasers, and policy makers to make informed decisions that will improve health care at the individual and population level [4]. Findings from effectiveness research are designed to strengthen health care systems by ensuring that care delivered is based on the best possible evidence and informed decisions. 

Nurses are optimally positioned to address many defined priorities in effectiveness science. These research priorities include testing the effectiveness of (1) primary prevention methods in preventing falls in older adults at varying degrees of risk; (2) dissemination and translation strategies to promote use of research findings by patients, clinicians, payers, and others; (3) strategies for reducing healthcare associated infections; (4) comprehensive care coordination programs in managing children and adults with chronic illness, especially in populations with known health disparities; (5) school based interventions involving meal programs, vending machines, and physical education, at different levels of intensity, in preventing and treating overweight and obesity in children and adolescents; and (6) various strategies (e.g., clinical interventions, social interventions) to prevent obesity, hypertension, diabetes, and heart disease in at-risk populations such as the urban poor and Native Americans/American Indians [4]. Nurse scientists have built and will continue to build programs of research in these and other important areas of need and must interface with the larger national and international effectiveness agenda to inform priorities for effectiveness science, and garner funds for their research. Additionally, nurse scientists have methodological expertise essential to move forward this national and international agenda; these include meta-analysis, research synthesis, large dataset analysis, and experimental and observational research methods. 

Our professional contributions to effectiveness science can best be achieved through unification models of education, practice, and research in which practice issues inform the effectiveness research agenda, researchers test interventions in real world settings, and practitioners, learners, and investigators can simultaneously understand why some healthcare interventions work in some settings, and with some populations, but not others. This deeper understanding of the mechanisms to improve health, patient outcomes, and healthcare systems will benefit consumers, payers, and public policy stakeholders. If we are to meet the healthcare challenges of today and into the future, our profession can no longer afford to isolate practice clinicians, educators and scientists and treat learners as guests in practice settings during their educational experience. We must unify to promote clinical inquiry, maximize the impact of research, promote excellence in patient care, and build a professional culture of life-long learning. 

## 2. Overview of Papers 

In this special edition, you will find papers related to different “views and levels” of the “clinical bridge,” processes to consider in building the bridge, some preliminary outcomes of unification in sample environments, as well as specific examples of the research that the faculty immersed in clinical environments can generate with application to clinical care.

### 2.1. Process of Partnership and Emerging Outcomes

Three papers focus on processes and outcomes of building the clinical bridge between academia and practice. The integrative review by J. A. Beal describes what is empirically known about academic-service partnerships in nursing. Analysis of publications revealed that empirical knowledge about academic-service partnerships is in the areas of prerequisites for successful partnerships, types and benefits of partnerships, and workforce development with themes of academic-practice progression and educational redesign. Most publications about academic service partnerships were descriptive in nature but did not include formal evaluation of outcomes. Future empirical work in this area should address the effectiveness of these partnerships using rigorous evaluation methods. 

Development of a robust clinical bridge in Australia, through jointly funded positions at the professorial level, is described by M. Wallis and W. Chaboyert. The evolution of this unique model of building a clinical bridge and expanding to several practice environments provides an exemplar for others interested in creating partnerships between practice and academia. The impact on research productivity, dissemination of research findings, and changes in practice are described. 

The paper by M. Svjeda and colleagues describe the process and outcomes of a partnership between the University of Michigan School of Nursing (UMSN) and nursing services at the University of Michigan Health System (UMHS) entitled “the Initiative for Excellence in Clinical Education, Scholarship and Practice.” The partnership is designed to leverage the resources and expertise of both organizations to advance the three missions of education, research, and practice. The whole-scale change process was used to bring faculty and practice stakeholders together for this initiative. An innovative model of clinical education was developed, piloted, and is now fully implemented in the undergraduate curriculum. Key findings of this model are provided. This paper addresses the processes, lessons learned, and successes of using an innovative method of education through building a clinical bridge between academia and practice. 

Collectively, these 3 papers point out the importance of evaluating these innovative academic-practice partnerships and lessons learned from these experiences. These exemplars are helpful to guide others interested in addressing the building of clinical bridges between academia and practice to maximize student learning, improving quality of care, and improving outcomes of learners. 

### 2.2. Context Issues and Effectiveness Science

Two studies focus on context factors in effectiveness science. In an effort to understand communication among clinicians, a key variable that impacts patient outcomes, D. Tschannen and E. Lee explored the impact of nursing characteristics (e.g., job category, education, experience, and expertise) on perceptions of communication in the acute care setting. Specifically, the more positive the environmental values (e.g., high trust, respect), the greater the perception of communication openness among nurses. They also found that nurses' experience was significantly related to openness of communication among nurses and physicians. Work environments that foster trust, respect, status equity, and time availability create an atmosphere where communication can flourish.

D. Wilson and colleagues compared the perceptions of nursing units' safety culture between charge nurses and staff nurses. The charge nurse was defined as a frontline nursing unit leader who makes shift-by-shift decisions about staffing, personnel, and unexpected events that impact patient care. They found that nurses with no charge experience had more positive overall perceptions of patient safety as compared to those with some charge experience. Charge nurses with one or more years of experience were less positive about teamwork, overall perceptions of safety, and number of safety events reported. These two papers draw attention to the importance of factors in the clinical environment, such as trust and characteristics of clinicians, that should be considered in conducting effectiveness science. 

### 2.3. Interventions and Technologies

Two papers address the effectiveness of interventions and technologies. L. L. Shever and M. G. Titler examined factors that contribute to adverse incidents of hospitalized older adults, using a model that included patient characteristics, clinical conditions, nursing unit context of care variables, medical treatments, pharmaceutical treatments, and nursing treatments. This exploratory observational study used data from electronic health records as well as other electronic repositories and demonstrated that the type of nursing treatments associated with adverse incidents were diverse. This study illustrates the importance of partnering between academic and practice sites that have electronic data to address nursing effectiveness research questions. 

P. A. Abbott addresses the challenges and opportunities in effective use of technology. This study evaluated how a specific technology (mobile clinical computing appliance consolidating numerous technological functions), designed to address technology crowding, is used in the real-world environment. Studies that compare how health IT is *actually used*, versus how the device was *designed to be used, *are necessary. This paper is a useful illustration of examining effectiveness of technology to assure usability and patient safety. 

### 2.4. Knowledge Application and Education

Three papers address innovations in knowledge application and education. A. Pearson and colleagues describe the nature of evidence-based healthcare and translation science, propose a reconceptualization that brings together these two conceptual areas, and asserts the existence of a third fundamental gap that is rarely addressed: the gap between knowledge need and discovery. They argue that if the evidence-based health care movement is to progress and make any real impact on health outcomes, it is imperative that the nature and processes of these two enterprises are integrated.

M. Aebersold and colleagues evaluate the effectiveness of virtual reality simulation, using two virtual simulation scenarios, to improve undergraduate student's performance regarding interpersonal skills (e.g., communication, professional behavior). The use of second life and creation of a virtual inpatient unit are among the innovative interventions used in this educational approach. Virtual world simulation environments offer a unique and potentially cost-effective method of teaching leadership and management skills. 

Moss and colleagues describe an innovative nursing research training model, the HOMInG Device, for doctoral education.This model and approach to research training focuses students on the interrelationships among four domains of their research enterprise—the substantive research topic, their interests as researchers, the research context, and particular research approach. Working with this model means that students engage consistently with these inter-relationships: from initial ideas and planning, to dissemination and positioning of the newly generated knowledge within clinical practice. Evaluative data suggest that this model facilitates doctoral education and research training. 

In summary, papers in this special issue are a source for generating greater understanding of how to proceed and what to expect along the path of development in building partnerships between academia and practice. The importance of such unified models provides benefits for effectiveness science, and application of innovative approaches to research, education, and practice. It should be noted that while these papers refer to work in hospital settings, the benefits of unification transcend specific environments and can and should benefit communities and populations with appropriate organizational partners.

*Kathleen Potempa* *Kathleen Potempa* *John Daly* *John Daly* *Marita G. Titler* *Marita G. Titler*
